# Predictive Factors Associated with Survival in Female Gastric Cancer Patients in Southeast Asia

**DOI:** 10.1089/whr.2023.0069

**Published:** 2024-02-23

**Authors:** Phubordee Bongkotvirawan, Natsuda Aumpan, Bubpha Pornthisarn, Soonthorn Chonprasertsuk, Sith Siramolpiwat, Patommatat Bhanthumkomol, Pongjarat Nunanun, Navapan Issariyakulkarn, Varocha Mahachai, Kammal Kumar Pawa, Ratha-korn Vilaichone

**Affiliations:** ^1^ Center of Excellence in Digestive Diseases and Gastroenterology Unit, Department of Medicine, Thammasat University, Pathumthani, Thailand.; ^2^ Department of Medicine, Chulabhorn International College of Medicine (CICM) at Thammasat University, Pathumthani, Thailand.

**Keywords:** gastric cancer, female, ASEAN, diffuse-type gastric cancer

## Abstract

**Introduction:**

Association of Southeast Asian Nations (ASEAN) countries have high *Helicobacter pylori* infections, and gastric cancer (GC) is a leading fatal cancer in this region, especially in female patients. This study aimed to compare clinical manifestations, histopathological subtypes, and prognostic factors associated with the overall survival rate of female GC patients in this important region.

**Methods:**

This retrospective cohort study was conducted between 2007 and 2022 at a tertiary care center in Thailand. All clinical information, endoscopic findings, and histological types were extensively reviewed. Furthermore, all qualified studies in ASEAN published in PubMed and Scopus between 2000 and 2022 were extracted and thoroughly analyzed. Young female GC patients are defined as those ≤50 years of age.

**Results:**

A total of 98 Thai female GC patients were included, with a mean age of 58.99 ± 14 years; 70.4% were elderly women. The common presenting symptoms were weight loss (69.4%) and dyspepsia (68.4%). Younger female GC patients had significantly more common diffuse-type GC than elderly female GC patients (82.8% vs. 53.6%, *p*-value = 0.007). Moreover, elderly female GC patients demonstrated significantly better survival than younger female GC patients (44.8% vs. 20.7%, odds ratio = 3.49; 95% confidence interval: 1.20–10.14, *p*-value = 0.022). Furthermore, a total of 1,491 female GC patients from ASEAN were reviewed and included in this study, aged 15 to 93 years. The top three countries with the highest proportion of female GC from ASEAN were Indonesia (66.7%), Thailand (44.9%), and Singapore (38.4%).

**Conclusion:**

GC in women is not uncommon in ASEAN and presents at an advanced stage with a grave prognosis. This study showed that ASEAN countries with the highest disease burden were Indonesia, Thailand, and Singapore. Overall, survival rates for female GC patients in ASEAN countries were relatively low, highlighting the need for proactive measures such as intensive *H. pylori* eradication and the development of early detection methods for GC.

## Introduction

Gastric cancer (GC) is a significant global health issue, with 1 million new cases and 760,000 deaths per year, notably in the Asia-Pacific area, including the Association of Southeast Asian Nations (ASEAN), where it has a high incidence and mortality rate. Despite the fact that men are approximately twice as likely as women to be affected by GC, it continues to be the fourth leading cause of cancer-related mortality among women worldwide and the seventh leading cause in ASEAN. GC continues to impact ∼16,000 women annually in ASEAN, with Singapore experiencing the highest prevalence, with a 5-year prevalence of 19.8 per 100,000 individuals.^1^ This may be affected by the region's high prevalence of *Helicobacter pylori* infection, ranging from 20% to ∼70%.^1,2^

Interestingly, men had a twofold higher incidence of GC than women^2,3^; this disparity disappears when women reach menopause.^4^ It is hypothesized that estrogen and progesterone produced during the menstrual cycle play a significant role as a protective factor against GC.^5^

Owing to the lack of available studies, information regarding prognostic factors and survival outcomes in women with GC is limited. This will be one of the pioneer studies to compare clinical manifestations, histopathological subtype differences, and survival prognostic factors between elderly and younger female GC patients in this region.

## Materials and Methods

### Study design

This retrospective cohort included all female GC patients at a tertiary care center in Thailand between August 2007 and August 2022. The absence of data as a potential source of bias was controlled by excluding patients with incomplete medical records. Demographic data, comorbidities, clinical manifestations, endoscopic features, GC location, *H. pylori* infection status, histopathological findings, staging according to the eighth edition AJCC cancer staging manual,^6^ treatment, and survival were all extracted and extensively reviewed from medical records.

An extensive review was conducted by searching Scopus and PubMed databases using the following terms: (“Gastric cancer” OR “Stomach cancer” AND “Female” OR “Women” OR “Woman” AND “ASEAN” OR “Thailand” OR “Singapore” OR “Vietnam” OR “Indonesia” OR “Cambodia” OR “Malaysia” OR “Brunei” OR “Myanmar” OR “Timor”) in English. The search was limited to articles containing data on patients with GC published before August 31, 2022. Two researchers evaluated and selected all relevant studies. None of the publications from Thailand utilized the same data as the patients from our center.

### Definitions

Younger female was defined as those whose chronological age is ≤50 years.

A diagnosis of GC was made by pathological diagnosis from a gastric biopsy.

Early-stage GC was defined as primary GC with an invasion depth no further than the submucosa, regardless of lymph node involvement. According to the eighth AJCC TNM staging,^6^ early GC is defined as T1 with any N.

### Statistical analysis

Continuous data are shown using the mean and standard deviation. Demographic data were analyzed by the chi-squared test or Fisher's exact test, whichever was appropriate. Univariate and multivariate analyses were performed to determine the relationship between variables. All tests were two sided, and a *p*-value <0.05 was regarded as statistically significant. IBM SPSS Statistics version 27.0 was used for the statistical analysis (SPSS, Inc., Armonk, NY).

This study adhered to the good clinical practice guidelines and the Declaration of Helsinki. Owing to the fact that this was a retrospective study with low risk and had no effect on the participant's well-being or rights, informed consent was waived. Only authors had access to the data, which were kept exclusively confidential. The obtained data are anonymous and cannot be used to identify specific individuals.

## Results

A total of 3,936 patients with GC from ASEAN were enrolled in this study. Within Thailand, 190 patients were included; among them, 98 (51.6%) were women. The mean age of female GC patients was 58.99 ± 14 years, and the majority (70.4%) were elderly GC patients. Common comorbidities in female GC patients were hypertension (26.5%), dyslipidemia (23.5%), and diabetes mellitus (19.4%). A subset of 28 patients (28.6%) had multiple comorbidities, and 66 patients (67.3%) had *H. pylori* infection.

The most prevalent presenting symptoms in female GC patients were weight loss (69.4%) and dyspepsia (68.4%). The majority of patients had diffuse-type GC (62.2%) and were in advanced stages (89.8%), with 60 patients (61.2%) having metastatic cancer. In addition, we included data from a literature review, incorporating an additional 3,746 GC patients into the total cohort of 3,936, of whom 1,491 (37.9%) were women.

Baseline patient demographic data, clinical characteristics, endoscopic findings, pathological subtype, and staging are demonstrated in Table 1.

**Table 1.** Baseline Characteristics of Younger and Elderly Female Gastric Cancer Patients

Characteristics	Younger women (***n*** = 29)	Elderly women (***n*** = 69)	***p*** ^b^
Mean age (years)	42.69 ± 6.62	65.84 ± 10.10	0.005
Comorbidities			
Hypertension	1/29 (3.4)^a^	25/69 (36.2)^a^	<0.001
Diabetes mellitus	2/29 (6.9)^a^	17/69 (24.6)^a^	0.043
Dyslipidemia	1/29 (3.4)^a^	22/69 (31.9)^a^	0.002
Chronic kidney disease	0/29 (0)^a^	6/69 (8.7)^a^	0.175
Multiple comorbidities	1/29 (3.4)^a^	27/69 (39.1)^a^	<0.001
History of smoking	2/29 (6.9)^a^	10/69 (14.5)^a^	0.501
History of alcohol drinking	16/29 (55.2)^a^	55/69 (79.7)^a^	0.013
Current *Helicobacter pylori* infection	17/29 (58.6)^a^	49/69 (71)^a^	0.232
Clinical manifestations			
Dyspepsia	18/29 (62.1)^a^	49/69 (71)^a^	0.385
Nausea/vomiting	11/29 (37.9)^a^	18/69 (26.1)^a^	0.241
Gastrointestinal bleeding	8/29 (27.6)^a^	20/69 (29)^a^	0.889
Anemia	12/29 (41.4)^a^	27/69 (39.1)^a^	0.836
Weight loss	20/29 (69)^a^	48/69 (69.6)^a^	0.953
Endoscopic findings			
Linitis plastica	6/29 (20.7)^a^	12/69 (17.4)^a^	0.700
Mass	8/29 (27.6)^a^	30/69 (43.5)^a^	0.141
Ulcer	7/29 (24.1)^a^	16/69 (23.2)^a^	0.919
Ulceroproliferative mass	8/29 (27.6)^a^	11/69 (15.9)^a^	0.183
Cancer type			
Diffuse type	24/29 (82.8)^a^	37/69 (53.6)^a^	0.007
Intestinal type	5/29 (17.2)^a^	32/69 (46.4)^a^	
Cancer staging			
Early stage	0/29 (0)^a^	10/69 (14.5)^a^	0.031
Advanced stage	29/29 (100)^a^	59/69 (85.5)^a^	
Treatment			
Surgery	8/29 (27.6)^a^	27/69 (39.1)^a^	0.276
Palliative chemotherapy	21/29 (72.4)^a^	34/69 (49.3)^a^	0.045
Concurrent chemoradiation	0/29 (0)^a^	6/69 (8.7)^a^	0.175
Targeted therapy	0/29 (0)^a^	2/69 (2.9)^a^	1.000
Survival			
1-year survival rate	6/29 (20.7)^a^	30/67 (44.8)^a^	0.025
5-year survival rate	1/29 (3.4)^a^	0/67 (0)^a^	0.302

^a^ *N* (column %).

^b^ Chi-square test for categorical variables.

### Primary outcome: Differences between gender, younger female group, and elderly female group

Men were found to be more likely to smoke than women (52.7% vs. 12.2%, *p*-value <0.001). Regarding clinical manifestation, women were more likely to present with dyspepsia and anemia than men (68.4% vs. 47.8%, *p*-value = 0.004, and 39.8% vs. 26.1%, *p*-value = 0.045, respectively). Moreover, women were found to have a higher incidence of diffuse-type GC than men (62.2% vs. 40.2%, *p*-value = 0.002). No significant differences were found in underlying diseases, endoscopic findings, and staging between the two genders.

All 98 female GC patients were divided into two groups by age. The elderly group consisted of 69 (70.4%) participants with a mean age of 65.84 ± 10.1 years, whereas the younger group had 29 (29.6%) participants with a mean age of 42.69 ± 6.62 years. Comorbidities are significantly more prevalent in the elderly group, including hypertension, diabetes mellitus, and dyslipidemia (36.2% vs. 3.4%, *p*-value <0.001, 24.6% vs. 6.9%, *p*-value = 0.043, and 31.9% vs. 3.4%, *p*-value = 0.002, respectively). The elderly group had more individuals with multiple comorbidities (39.1% vs. 3.4%, *p*-value <0.001). In addition, the elderly group was also more likely to have a history of alcohol consumption than the younger group (79.7% vs. 55.2%, *p*-value = 0.013).

Regarding the histopathology, even though there was more diffuse-type GC in both groups, the younger group had a significantly higher prevalence of diffuse-type GC (82.8% vs. 53.6%, *p*-value = 0.007). Moreover, the younger group had a higher likelihood of presenting with advanced-stage GC (100% vs. 85.5%, *p*-value = 0.031). There was no significant difference in terms of clinical manifestations, endoscopic features, location of GC, and site of metastasis between the two age groups.

### Survival outcomes and prognostic factors associated with mortality

Of the 190 GC patients, 183 were included in the survival analysis after attending follow-up visits, whereas 7 were excluded due to being lost to follow-up. Of the seven patients, five were men, and two were women, both being in the elderly female GC group. The overall 1-, 2-, and 5-year survival rates in female GC patients were 37.5%, 13.5%, and 1%, respectively. Female GC patients had better 1-year survival rates and 2-year survival rates than male GC patients (37.5% vs. 23%, *p*-value = 0.033, and 13.5% vs. 1.1%, *p*-value = 0.002, respectively).

**FIG. 1.**
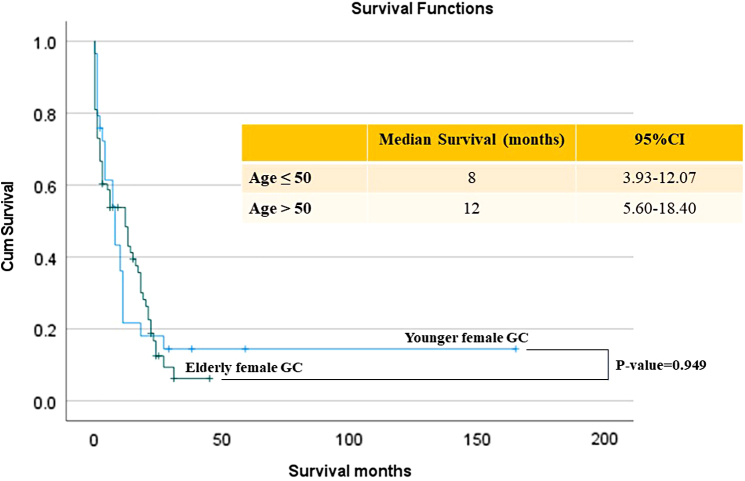
Survival analysis of female GC patients in this study. ASEAN, Association of Southeast Asian Nations; CCRT, concurrent chemoradiotherapy; CMT, chemotherapy; GC, gastric cancer; HDGC, hereditary diffuse gastric cancer; N/A, not applicable; OS, overall survival.

Elderly female GC patients had significantly better 1-year survival rates than the younger female GC group (44.8% vs. 20.7%, odds ratio [OR] = 3.49; 95% confidence interval [CI]: 1.20–10.14; *p*-value = 0.022). However, Kaplan–Meier survival analysis revealed that there was no significant difference in median survival between elderly and younger female GC patients, as shown in Figure 1.

Regarding prognostic factors associated with mortality, several statistically significant univariate variables were analyzed in a Cox multivariate analysis. Younger female GC group (OR = 3.49; 95% CI: 1.20–10.14; *p*-value = 0.022) and diffuse-type GC group (OR = 3.50; 95% CI: 1.45–8.45; *p*-value = 0.005) were significantly associated with mortality. Univariate and multivariate analyses of prognostic factors associated with mortality are demonstrated in Table 2.

**Table 2.** Cox Multivariate Analysis of Predictive Factors for Mortality of Female Gastric Cancer

Variables	Univariate analysis			Multivariate analysis		
	OR^a^	95% CI	***p***	OR	95% CI	***p***
Age ≤50	2.95	1.07–8.15	0.03	3.49	1.20–10.14	0.022
Diabetes	0.99	0.35–2.81	0.991	1.12	0.17–1.86	0.869
Hypertension	0.88	0.35–2.26	0.794	1.05	0.22–1.61	0.305
Diffuse-type GC	3.31	1.40–7.84	0.007	3.50	1.45–8.45	0.005
Advanced stage cancer	1.84	0.49–6.85	0.364	0.62	0.15–2.49	0.499

^a^ Cox multivariate analysis adjusting from all significant variables from univariate analysis, including age ≤50 and diffuse-type GC.

CI, confidence interval; GC, gastric cancer; OR, odds ratio.

### Female GC in ASEAN

Fourteen studies involving patients with GC were reported from seven different countries of ASEAN (Thailand, Brunei, Malaysia, Vietnam, Singapore, Indonesia, and the Philippines). There were 3,936 cases of GC, with 1,491 (37.9%) being women. The highest proportion of female GC patients was observed in Indonesia (66.7%), followed by Thailand (44.9%) and Singapore (38.4%), whereas the lowest proportion of female GC patients was found in the Philippines (22.2%). The mean age of female patients ranged from 58.4 ± 14.8 to 58.99 ± 14 years old.

The prevalence of diffuse-type GC varied widely across studies, ranging from 29.8% to 52.6%. Most patients were diagnosed at an advanced stage, with the majority being diagnosed at stage 4, ranging from 8.8% to 57.1%. Consequently, the mortality rates were high, with 1- and 5-year survival rates ranging from 37.5% to 46.2% and 1% to 17.8%, respectively. Chemotherapy was the most common treatment option, received by 56.1% to 63.8% of patients, followed by surgery (20.7% to 35.7%). Data from Cambodia, Laos, Myanmar, and Timor-Leste were not available. All clinical characteristics and survival rates of male and female GC patients in ASEAN are demonstrated in Table 3, and those exclusively of female GC patients in Table 4.

**Table 3.** Clinical Characteristics and Survival of Female and Male Gastric Cancer Patients in Association of Southeast Asian Nations

Study (year)	Country	Total patients, %F	Mean age (years)	Diffuse type (%)	Cancer staging	Overall survival
This study	Thailand	190/51.6%	60.5 ± 14.2	Diffuse type 51.6%	Advanced stage (91.5%)	1-year survival rate = 30.6% 2-year survival rate = 7.7% 5-year survival rate = 0.5%
Nanthanangkul et al. (2016)^19^	Thailand	650/43.9%	N/A	N/A	Advanced stage (49.85%)	1-year survival rate = 32.2% 2-year survival rate = 23.8% 5-year survival rate = 17.2%
Punjachaipornpon et al. (2016)^20^	Thailand	53/49.1%	60.6	Diffuse type 26.4%	Advanced stage (62.3%)	1-year survival rate = 77.4% 5-year survival rate = 17.0%
Poonyam et al. (2021)^21^	Thailand	100/43%	61.1	Diffuse type 38%	Advanced stage (92%)	1-year survival rate = 48.9%
Chong et al. (2014)^22^	Brunei	572/35.7%	60.43 ± 16.1	N/A	N/A	N/A
Kandasami et al. (2003)^23^	Malaysia	250/31.6%	63.2	N/A	Advanced stage (96.4%)	N/A
Goh et al. (2007)^24^^)^	Malaysia	87/51.7%	61.4 ± 13.0	Diffuse type 32%	N/A	N/A
Rajadurai et al. (2018)^25^	Malaysia	228/37.7%	60.3 ± 13.7	Diffuse type 41.2%	Advanced stage (81.6%)	N/A
Binh et al. (2017)^26^	Vietnam	282/20.2%	62.5 ± 12.6	Diffuse type 55.3%	N/A	N/A
Dang et al. (2019)^27^	Vietnam	182/31.9%	60.8 ± 11.6	Diffuse type 24.6%	Advanced stage (91.8%)	N/A
Chuwa et al. (2005)^28^	Singapore	142/35.9%	67.2	N/A	Advanced stage (74.6%)	N/A
Tan et al. (2019)^29^	Singapore	470/43.8%	64.8 ± 13.4	Diffuse type 47.2%	Advanced stage (100%)	N/A
Lui et al. (2019)^30^	Singapore	405/33.1%	N/A	Diffuse type 30.6%	Advanced stage (81.5%)	N/A
Warsinggih et al. (2022)^31^	Indonesia	54/66.7%	N/A	Diffuse type 4.7%	N/A	N/A
Pathmanathan et al. (2017)^32^	Malaysia	6/33.3%	N/A	Diffuse type 16.6%	N/A	N/A ^*^Multicenter study
	Philippines	9/22.2%				
	Vietnam	204/30.4%				
	Thailand	52/32.7%				

%F, female; N/A, nonapplicable.

**Table 4.** Summary of Clinical Characteristics and Survival of Female Gastric Cancer Patients in Association of Southeast Asian Nations

Study (year)	Country	Total patient, %F	Diffuse type (%)	Cancer staging	Treatment	Overall survival
This study	Thailand	190/51.6%	Diffuse type 51.6%	Stage 1 = 10.2% Stage 2 = 5.1% Stage 3 = 27.6% Stage 4 = 57.1%	Palliative CMT = 56.1% Surgery = 35.7% CCRT = 6.1% Targeted therapy = 2%	1-year survival rate = 37.5% 2-year survival rate = 13.5% 5-year survival rate = 1%
Nanthanangkul et al. (2016)^19^	Thailand	650/43.9%	N/A	N/A	N/A	5-year survival rate = 17.8%
Poonyam et al. (2021)^21^	Thailand	100/43%	Diffuse type 46.5%	Stage 1 = 2.3% Stage 2 = 7% Stage 3 = 41.9% Stage 4 = 48.8%	N/A	1-year survival rate = 46.2%
Binh et al. (2017)^26^	Vietnam	282/20.2%	Diffuse type 52.6%	N/A	N/A	N/A
Dang et al. (2019)^27^	Vietnam	182/31.9%	Diffuse type 29.8%	Stage 1 + 2 = 32.4% Stage 3 = 14.7% Stage 4 = 8.8% Not defined = 44.1%	Surgery = 20.7% CMT+surgery = 63.8% Others = 15.5%	N/A

CCRT, concurrent chemoradiotherapy; CMT, chemotherapy; OS, overall survival.

## Discussion

GC is the fifth most common cancer and the fourth leading cause of cancer-related death worldwide. Approximately 16,000 new cases of female GC are annually diagnosed in the region.^1^ This study demonstrated a comparable ratio of male-to-female patients with GC, in contrast to the predominance of male patients in all other ASEAN studies. The male predominance in GC incidence may not solely be due to the protective effect of estrogen in women. Other factors, such as tobacco use, variations in dietary patterns, and occupational exposure, may also contribute to the increased prevalence of GC among men.^7^

Moreover, this study revealed that the majority of female GC patients were elderly and diagnosed at an advanced stage, which was comparable with previous studies,^2,8–21^ notably that all of the younger group presented at an advanced stage. This appears to be comparable with a study from China.^8^ Furthermore, this study demonstrated a significantly higher proportion of diffuse-type GC in the younger group. Estrogen has been proposed to play a role in the development of diffuse-type GC. A prior study has reported an elevated positivity rate of estrogen receptors (ERs) and tumorigenic mechanism of estrogen in the development of ER-positive diffuse-type GC in young female patients, which may account for the higher incidence of this subtype among younger female GC patients.^9^

Moreover, our study demonstrates that having diffuse-type GC and being younger have a lower survival rate. This is consistent with previous studies indicating that patients with young-onset GC and diffuse-type GC had a poor prognosis.^8,10,11^ This could be attributed to the absence of precancerous lesions associated with diffuse-type GC, resulting in challenges in early detection, leading to late presentation and diagnosis at a more advanced stage of the disease. Furthermore, *CDH1* gene mutations may also contribute to late diagnosis.

These mutations can disrupt gastric cell polarity, causing the loss of the epithelial cell adhesion protein E-cadherin, and result in diffuse-type GC without atrophic chronic gastritis or intestinal metaplasia. This can lead to hereditary diffuse GC, with an incidence of ∼5–10/100,000 births.^12^ These mutations are more commonly found in younger patients,^13^ resulting in delayed diagnosis, rapid tumor growth, and poor treatment response. This interplay of factors can contribute to a lower 1-year survival rate among younger female GC patients.

Our study compiled and analyzed 14 studies with GC patients from 7 countries throughout ASEAN. The proportion of women with GC in comparison with men with GC ranges from 22.2% to 66.7%. Among the countries analyzed, Indonesia has the highest proportion at 66.7%, followed by Thailand at 44.9% and Singapore at 38.4%. The lowest proportion was found in the Philippines at 22.2%. This contradicts the GLOBOCAN report for 2020, which indicates that the countries with the highest incidence of GC over a 5-year period are Vietnam, Brunei Darussalam, and Singapore.^1^

The disparity between the findings of this study and GLOBOCAN might be attributed to differences in some factors such as number of studies conducted, study period, and population. Furthermore, this study gained more information and defined current knowledge on the incidence of GC among women in ASEAN, especially Thailand. Singapore had a high prevalence of GC among ASEAN, and Singapore's incidence rate of 38.4% reported in this study is consistent with a report from GLOBOCAN.

Therefore, Singapore remains a country of concern with growing problems with female GC. Furthermore, our study demonstrated that female diffuse-type GC is more common among both elderly and younger female GC patients and that the majority of patients were in advanced stages. This is comparable with studies from other regions in Asia, such as East Asia,^10,14^ the Middle East,^15^ and Europe.^16^

Effective screening programs, early diagnosis, and treatment of GC in young female patients may play a pivotal role in improving survival rates and treatment outcomes. Many systematic reviews and meta-analyses concluded that eradication of *H. pylori* in infected adults reduced the risk of developing GC.^17,18^ Although the optimal screening system for this particular patient population is still unknown.

## Conclusion

In conclusion, GC in female patients remains a major concern in ASEAN, and differences in clinical characteristics and histopathological findings between age groups can affect prognosis. Younger female patients with diffuse-type GC are associated with higher mortality rates. This underscores the crucial need for proactive measures, including comprehensive *H. pylori* eradication and the development of effective screening and early detection methods for GC.

## Data Availability

The deidentified data sets collected and analyzed during this study are available from the corresponding authors upon reasonable request.

## Abbreviations Used

ASEAN: Association of Southeast Asian Nations
CCRT: concurrent chemoradiotherapy
CI: confidence interval
CMT: chemotherapy
ERs: estrogen receptors
GC: gastric cancer
HDGC: hereditary diffuse gastric cancer
N/A: nonapplicable
OR: odds ratio
OS: overall survival

## References

[1] Sung H, Ferlay J, Siegel RL, et al. Global Cancer Statistics 2020: GLOBOCAN Estimates of Incidence and Mortality Worldwide for 36 Cancers in 185 Countries. CA Cancer J Clin 2021;71(3):209–249; doi: 10.3322/caac.21660 33538338

[2] Ang TL, Fock KM. Clinical epidemiology of gastric cancer. Singapore Med J 2014;55(12):621–628; doi: 10.11622/smedj.2014174 25630323 PMC4291998

[3] Ferlay J, Colombet M, Soerjomataram I, et al. Cancer statistics for the year 2020: An overview. Int J Cancer 2021;149(4):778–789; doi: 10.1002/ijc.33588 33818764

[4] Lou L, Wang L, Zhang Y, et al. Sex difference in incidence of gastric cancer: An international comparative study based on the Global Burden of Disease Study 2017. BMJ Open 2020;10(1):e033323; doi: 10.1136/bmjopen-2019-033323 PMC7044958 31988231

[5] Freedman ND, Chow WH, Gao YT, et al. Menstrual and reproductive factors and gastric cancer risk in a large prospective study of women. Gut 2007;56(12):1671–1677; doi: 10.1136/gut.2007.129411 17627962 PMC2095686

[6] Amin MB, Greene FL, Edge SB, et al. The Eighth Edition AJCC Cancer Staging Manual: Continuing to build a bridge from a population-based to a more “personalized” approach to cancer staging. CA Cancer J Clin 2017;67(2):93–99; doi: 10.3322/caac.21388 28094848

[7] Rawla P, Barsouk A. Epidemiology of gastric cancer: Global trends, risk factors and prevention. Prz Gastroenterol 2019;14(1):26–38; doi: 10.5114/pg.2018.80001 30944675 PMC6444111

[8] Cheng L, Chen S, Wu W, et al. Gastric cancer in young patients: A separate entity with aggressive features and poor prognosis. J Cancer Res Clin Oncol 2020;146(11):2937–2947; doi: 10.1007/s00432-020-03268-w 32451690 PMC11804640

[9] Kang S, Park M, Cho JY, et al. Tumorigenic mechanisms of estrogen and *Helicobacter pylori* cytotoxin-associated gene A in estrogen receptor α-positive diffuse-type gastric adenocarcinoma. Gastric Cancer 2022;25(4):678–696; doi: 10.1007/s10120-022-01290-0 35391613

[10] Kim HW, Kim JH, Lim BJ, et al. Sex disparity in gastric cancer: Female sex is a poor prognostic factor for advanced gastric cancer. Ann Surg Oncol 2016;23(13):4344–4351; doi: 10.1245/s10434-016-5448-0 27469120

[11] Kono Y, Kanzaki H, Iwamuro M, et al. Reality of gastric cancer in young patients: The importance and difficulty of the early diagnosis, prevention and treatment. Acta Med Okayama 2020;74(6):461–466; doi: 10.18926/amo/61204 33361865

[12] Liu X, Chu KM. E-cadherin and gastric cancer: Cause, consequence, and applications. Biomed Res Int 2014;2014:637308; doi: 10.1155/2014/637308 25184143 PMC4145387

[13] Cho SY, Park JW, Liu Y, et al. Sporadic early-onset diffuse gastric cancers have high frequency of somatic CDH1 alterations, but low frequency of somatic RHOA mutations compared with late-onset cancers. Gastroenterology 2017;153(2):536–549; doi: e26.10.1053/j.gastro.2017.05.012 28522256 10.1053/j.gastro.2017.05.012 PMC6863080

[14] Choi Y, Kim N, Kim KW, et al. Sex-based differences in histology, staging, and prognosis among 2983 gastric cancer surgery patients. World J Gastroenterol 2022;28(9):933–947; doi: 10.3748/wjg.v28.i9.933 35317055 PMC8908285

[15] Bani-Hani KE, Yaghan RJ, Heis HA, et al. Gastric malignancies in Northern Jordan with special emphasis on descriptive epidemiology. World J Gastroenterol 2004;10(15):2174–2178; doi: 10.3748/wjg.v10.i15.2174 15259060 PMC4724987

[16] Jaehn P, Holleczek B, Becher H, et al. Histologic types of gastric cancer among migrants from the former Soviet Union and the general population in Germany: What kind of prevention do we need? Eur J Gastroenterol Hepatol 2016;28(8):863–870; doi: 10.1097/meg.0000000000000645 27187801

[17] Ford AC, Yuan Y, Moayyedi P. *Helicobacter pylori* eradication therapy to prevent gastric cancer: Systematic review and meta-analysis. Gut 2020;69(12):2113–2121; doi: 10.1136/gutjnl-2020-320839 32205420

[18] Lee YC, Chiang TH, Chou CK, et al. Association between *Helicobacter pylori* eradication and gastric cancer incidence: A systematic review and meta-analysis. Gastroenterology 2016;150(5):1113–1124.e5; doi: 10.1053/j.gastro.2016.01.028 26836587

[19] Nanthanangkul S, Suwanrungruang K, Wiangnon S, et al. Survival of stomach cancer cases in Khon Kaen, Thailand 2000–2012. Asian Pac J Cancer Prev 2016;17(4):2125–2129; doi: 10.7314/apjcp.2016.17.4.2125 27221906

[20] Punjachaipornpon T, Mahachai V, Vilaichone R. Severe manifestations and grave prognosis in young patients with gastric cancer in Thailand. Asian Pac J Cancer Prev 2016;17(7):3427–3429. 27509987

[21] Poonyam P, Aumpan N, Vilaichone RK. Prognostic factors for survival in patients with gastric adenocarcinoma. Cancer Rep (Hoboken) 2021;4(1):e1305; doi: 10.1002/cnr2.1305 33074592 PMC7941448

[22] Chong VH, Telisinghe PU, Abdullah MS, et al. Gastric cancer in Brunei Darussalam: Epidemiological trend over a 27 year period (1986–2012). Asian Pac J Cancer Prev 2014;15(17):7281–7285; doi: 10.7314/apjcp.2014.15.17.7281 25227829

[23] Kandasami P, Tan WJ, Norain K. Gastric cancer in Malaysia: The need for early diagnosis. Med J Malaysia 2003;58(5):758–762. 15190664

[24] Goh KL, Cheah PL, Md N, et al. Ethnicity and *H. pylori* as risk factors for gastric cancer in Malaysia: A prospective case control study. Am J Gastroenterol 2007;102(1):40–45; doi: 10.1111/j.1572-0241.2006.00885.x 17100981

[25] Rajadurai P, Fatt HK, Ching FY. Prevalence of HER2 positivity and its clinicopathological correlation in locally advanced/metastatic gastric cancer patients in Malaysia. J Gastrointest Cancer 2018;49(2):150–157; doi: 10.1007/s12029-017-9921-1 28124769 PMC5948243

[26] Binh TT, Tuan VP, Dung HDQ, et al. Advanced non-cardia gastric cancer and *Helicobacter pylori* infection in Vietnam. Gut Pathog 2017;9:46; doi: 10.1186/s13099-017-0195-8 28824711 PMC5561603

[27] Ngoc Thi Dang D, Ngoc Thi Nguyen L, Thi Dang N, et al. Quality of life in vietnamese gastric cancer patients. Biomed Res Int 2019;2019:7167065; doi: 10.1155/2019/7167065 31236411 PMC6545786

[28] Chuwa EW, Khin LW, Chan WH, et al. Prognostic significance of peritoneal lavage cytology in gastric cancer in Singapore. Gastric Cancer 2005;8(4):228–237; doi: 10.1007/s10120-005-0343-6 16328597

[29] Tan HL, Chia CS, Tan GHC, et al. Metastatic gastric cancer: Does the site of metastasis make a difference? Asia Pac J Clin Oncol 2019;15(1):10–17; doi: 10.1111/ajco.13025 29920947

[30] Lui SA, Tan WB, Tai BC, et al. Predictors of survival outcome following radical gastrectomy for gastric cancer. ANZ J Surg 2019;89(1–2):84–89; doi: 10.1111/ans.15011 30690932

[31] Warsinggih, Syarifuddin E, Marhamah, et al. Association of clinicopathological features and gastric cancer incidence in a single institution. Asian J Surg 2022;45(1):246–249; doi: 10.1016/j.asjsur.2021.05.004 34090784

[32] Pathmanathan N, Geng JS, Li W, et al. Human epidermal growth factor receptor 2 status of gastric cancer patients in Asia: Results from a large, multicountry study. Asia Pac J Clin Oncol 2017;13(3):249–260; doi: 10.1111/ajco.12653 28008715

